# A DNA Methylation-Based Gene Signature Can Predict Triple-Negative Breast Cancer Diagnosis

**DOI:** 10.3390/biomedicines9101394

**Published:** 2021-10-04

**Authors:** Saioa Mendaza, David Guerrero-Setas, Iñaki Monreal-Santesteban, Ane Ulazia-Garmendia, Alicia Cordoba Iturriagagoitia, Susana De la Cruz, Esperanza Martín-Sánchez

**Affiliations:** 1 Molecular Pathology of Cancer Group, Navarrabiomed, Complejo Hospitalario de Navarra (CHN), Universidad Pública de Navarra (UPNA), Instituto de Investigación Sanitaria de Navarra (IdiSNA), Irunlarrea 3, 31008 Pamplona, Spain; dguerres@navarra.es (D.G.-S.); imonreas@navarra.es (I.M.-S.); ane.ulazia.garmendia@gmail.com (A.U.-G.); 2 Department of Pathology, Complejo Hospitalario de Navarra (CHN), Irunlarrea 3, 31008 Pamplona, Spain; alicia.cordoba.iturriagagoitia@navarra.es; 3 Department of Medical Oncology, Complejo Hospitalario de Navarra (CHN), Irunlarrea 3, 31008 Pamplona, Spain; sdelacrs@navarra.es

**Keywords:** DNA methylation, diagnosis, epigenetic biomarker, diagnosis signature, triple-negative breast cancer

## Abstract

Triple-negative breast cancer (TNBC) is the most aggressive breast cancer (BC) subtype and lacks targeted treatment. It is diagnosed by the absence of immunohistochemical expression of several biomarkers, but this method still displays some interlaboratory variability. DNA methylome aberrations are common in BC, thereby methylation profiling could provide the identification of accurate TNBC diagnosis biomarkers. Here, we generated a signature of differentially methylated probes with class prediction ability between 5 non-neoplastic breast and 7 TNBC tissues (error rate = 0.083). The robustness of this signature was corroborated in larger cohorts of additional 58 non-neoplastic breast, 93 TNBC, and 150 BC samples from the Gene Expression Omnibus repository, where it yielded an error rate of 0.006. Furthermore, we validated by pyrosequencing the hypomethylation of three out of 34 selected probes (*FLJ43663*, PBX Homeobox 1 (*PBX1*), and RAS P21 protein activator 3 (*RASA3*) in 51 TNBC, even at early stages of the disease. Finally, we found significantly lower methylation levels of *FLJ43663* in cell free-DNA from the plasma of six TNBC patients than in 15 healthy donors. In conclusion, we report a novel DNA methylation signature with potential predictive value for TNBC diagnosis.

## 1. Introduction

Breast cancer (BC) has overtaken lung cancer as the most commonly diagnosed cancer globally in 2020 and is the top cause of deaths from cancer for women worldwide, becoming a public health major issue [1]. BC’s heterogeneity is reflected in its division into several molecular subtypes with a variety of pathological features, leading to diverse treatment options and prognoses. This categorisation relies on the differential expression of key genes in tumour initiation and progression [2]. As a consequence, current methods for BC subtype identification are based on the immunohistochemical expression of oestrogen receptor (ER), progesterone receptor (PR), and Ki-67, along with the expression and/or amplification of the gene encoding the human epidermal growth factor 2 receptor (HER2) [3]. Based on these biomarkers, BC can be classified into five intrinsic subtypes: Luminal A (ER+, PR+, HER2-, low Ki-67), Luminal B/HER2-negative (ER+, PR-/+, HER2-, low Ki-67), Luminal B/HER2-positive (ER+, PR-, HER2+, high Ki-67; or ER+, PR+, HER2+, low Ki-67), HER2-positive (ER-, PR-, HER2+, any Ki-67), and triple-negative BC (TNBC) (ER-, PR-, HER2-, any Ki-67) [4]. Besides diagnosis, these biomarkers have been shown to be suitable targets for targeted therapy, such as hormone therapy for luminal subtypes and anti-HER2 antibodies for HER2-overexpressing subtypes. Since TNBC is characterized by the lack of ER and PR expression, combined with the absence of both overexpression and amplification of the gene encoding HER2 [2,5], the targeted treatment that is effective in the other BC subtypes is futile for TNBC patients [6]. Thus, TNBC, which accounts for about 15% of all BC cases, is considered the most aggressive subgroup portrayed by early relapse, frequent distant metastasis, and poor overall survival [7]. Despite its malignancy, TNBC diagnosis remains a major concern worldwide [8], because it still relies on the subjective evaluation of immunostaining assays that are not 100% sensitive and specific [9], and show considerable interlaboratory variability [10]. Apart from immunohistochemistry, the development of other diagnosis methods has started for BC. Some investigations have been performed to identify gene-expression and epigenetic alterations as diagnosis biomarkers, including blood-based markers for non-invasive detection [11,12,13]. However, this field is poorly explored in TNBC. Therefore, a more reliable assessment based on additional molecular and quantifiable biomarkers is highly desirable [10,14].

Aberrations in DNA methylation patterns are known to be crucial players in cancer initiation [15,16]. Hence, DNA methylation signatures are being assessed as potential molecular biomarkers in cancer [17,18,19], and specifically, several DNA-methylation based biomarkers have been described to have significant diagnostic and prognostic potential in BC [15,20]. Remarkably, since DNA-methylation changes can also be detected in biological fluids, some of these biomarkers can be tracked in cell-free DNA (cfDNA) from BC patients’ plasma [21,22,23], which makes them really attractive from the translational point of view. However, few DNA-methylation alterations have been reported in TNBC [24,25,26,27,28], and nearly all of them have been proposed to have predictive value of prognosis and drug response, but not as diagnostic biomarkers. Thus, in this study, our aim was to identify a DNA-methylation-based diagnostic signature to establish new supplementary diagnostic tools for TNBC detection.

## 2. Materials and Methods

### 2.1. DNA Methylome Data Sets

In this study, we used seven data sets deposited in Gene Expression Omnibus (GEO, https://www.ncbi.nlm.nih.gov/geo/ assessed on 10 June 2019). All of them reported DNA methylation data profiled using the Illumina Infinium Methylation 450K BeadChip assay (Illumina, San Diego, CA, USA), which made them easily comparable. The signature was constructed from one of these data sets (GSE141338), generated by our group in a previous study [28], using non-neoplastic mammary tissues from 6 reduction mammoplasties, and tumours from 8 patients with TNBC. The remaining data sets, which were used to confirm the signature, included DNA methylation profiles from 58 non-neoplastic breast samples (GSE88883 [29] and GSE74214), 93 TNBC (GSE78751 [30] and GSE78754 [29]), 150 BC other than TNBC (GSE141338 [28] and GSE72245 [31]), and 30 non-neoplastic prostate and 30 prostate cancer samples (GSE76938).

### 2.2. Bioinformatics Analysis

#### 2.2.1. Generation of a Diagnostic Signature for TNBC

With the aim of finding a signature with diagnostic potential for TNBC, we identified differentially methylated probes (DMPs) between six non-neoplastic mammary and eight TNBC tissues, with class prediction ability in our discovery data set. First, the methylation level of each of the 450,000 CpG sites interrogated in the array was estimated as normalized β values using the GenomeStudio program v2010.3 (Illumina, San Diego, CA, USA). β values ranged between 0 (fully unmethylated) and 1 (fully methylated). Thereafter, and without filtering by methylation fold change between TNBC and non-neoplastic tissues (Δβ) or genomic region, all 450,000 probes were subjected to a class prediction algorithm based on the K-nearest neighbours (KNN) method, and predictive probes were selected using the ANOVA F-ratio using the Tnasas tool (http://tnasas.iib.uam.es/ assessed on 20 June 2019). Briefly, KNN is a non-parametric analysis that predicts the samples of a test case as the majority vote among its *k* nearest neighbours. These ones are chosen based on the Euclidean distance, and their number (*k*) is selected by cross-validation. For probe selection, the program divided the whole series in 10 subsets and ranked the probes using ANOVA F-ratio, which provided a first set of ranked probes to further feed the predictor. The predictor was then built using different numbers of the best-ranked probes, and the left-out sample was predicted for each of them. The cross-validation error corresponding to each number of probes was computed, and the best predictor set was the one with the smallest cross-validation error and the smallest number of probes. Finally, the program run the probe-ranking method on the complete series and selected the top probes. To evaluate the error rate, the program divided again the whole series in 10 subsets, and for each of them, left aside one (the “out-of-bag” subset), found the best number of probes with the other 9 subsets (the “in-bag” ones), as just described, and predicted the out-of-bag samples with the predictor just found. Since at the end of the process, each sample was once in the “out-of-bag” set, the final error rate was obtained using all out-of-bag predictions. As the final output, the predictor set with the smallest error rate was returned.

#### 2.2.2. Robustness of the Model

The potential of the signature was then assessed by applying its predictive power into larger cohorts. To do this, the methylation levels of the predictive probes were retrieved from data sets publicly available in the GEO repository, particularly, of non-neoplastic breast (*n* = 58) and TNBC samples (*n* = 93). Moreover, in order to assure whether these selected DMPs predicted TNBC specifically or were related to any type of BC, their methylation levels were interrogated in additional series of breast tumours not belonging to the TNBC subtype (*n* = 150). Furthermore, data from an unrelated-to-breast tissue (30 non-neoplastic prostate and 30 prostate cancer samples) were explored to test the accuracy of those probes as classifiers and therefore TNBC diagnostic biomarkers.

Finally, unsupervised clusterings were performed with the Babelomics 5 tool (http://babelomics.bioinfo.cipf.es/ assessed on 15 July 2019) [32], using the unweighted pair group method with arithmetic mean (UPGMA) method and the normal Euclidean distance.

### 2.3. Validation of the Signature

#### 2.3.1. Patient Samples

Two cohorts of patients were used in this study to confirm the predictive diagnostic value of the signature. First, a series of formalin-fixed, paraffin-embedded (FFPE) samples from 51 patients with TNBC and 57 with BC of distinct subtypes other than TNBC, and 16 non-neoplastic breast tissues from reduction mammoplasties, was employed to validate the DNA methylation signature. Lastly, the methylation status of selected genes belonging to the signature was explored in cfDNA in a small series of plasma samples from six TNBC patients and 15 age-matched healthy women. 

All patients were diagnosed with invasive ductal breast carcinoma in the Department of Pathology (Complejo Hospitalario de Navarra, Pamplona, Spain) in accordance with the criteria recommended by the St Gallen International Expert Consensus 2013 [4], considering specific Ki-67 threshold [33], grading according to the Nottingham system [34], and staging based on the AJCC (American Joint Committee on Cancer) system [35]. All cancer tissue samples harboured at least 70% of tumour cells. None of the patients had received radiotherapy or chemotherapy before surgery. Their pathological and clinical characteristics are summarised in Supplementary Table S1.

#### 2.3.2. DNA and cfDNA Extraction and Bisulphite Conversion

To assess DNA methylation status, DNA and total cfDNA were extracted from cancer patients’ and healthy women’s mammary tissue and plasma samples using the QIAamp DNA FFPE Tissue kit and the QIAamp Circulating Nucleic Acid Kit (both from Qiagen, Hilden, Germany), respectively, and following the manufacturer’s instructions. After quantifying DNA concentration and purity in a NanoDrop spectrophotometer (Thermo Scientific, Waltham, MA), bisulphite conversion of 500 ng of DNA or 100 ng of cfDNA was performed using the EZ DNA Methylation-Gold kit (Zymo Research, Irvine, CA, USA) following the manufacturer’s recommendations.

#### 2.3.3. Pyrosequencing

To confirm the methylation levels of selected genes, pyrosequencing was performed in bisulphite-converted DNA from FFPE tissues (51 TNBC, 57 BC of other subtypes and 16 non-neoplastic mammary tissues), and cfDNA from plasma (6 TNBC patients and 15 healthy donors). First, 2 μL of bisulphite-modified DNA or cfDNA were amplified by PCR using 0.5 μL Immolase DNA polymerase (BioLine, London, UK) in a final volume of 30 μL, and with the primers which amplified the same region recognized by the probe contained in the array (Table 1). Amplification conditions consisted of an initial DNA polymerase activation at 95 °C for 10 min, followed by 50 cycles at 95 °C for 30 s, specific melting temperature for each gene (Table 1) for 30 s and 72 °C for 30 s, and a final extension at 72 °C for 7 min. Then, pyrosequencing was carried out in a PyroMark q96 (Qiagen, Hilden, Germany) as previously described [36].

### 2.4. Statistical Analysis

Demographic, clinical, and pathological data were summarised as frequencies (and percentages) or means (and ranges), as appropriate. Medians of methylation in tumour and non-neoplastic tissues were compared using the Mann–Whitney U test. The optimal cut-off value identifying the hypomethylated or hypermethylated status of each selected probes measured by pyrosequencing was estimated by ROC curve analyses. Across several cut-off points, the largest positive likelihood ratio was chosen as the optimal value [37].

## 3. Results

### 3.1. Novel Diagnostic DNA Methylation Signature for TNBC

**Figure 1:**
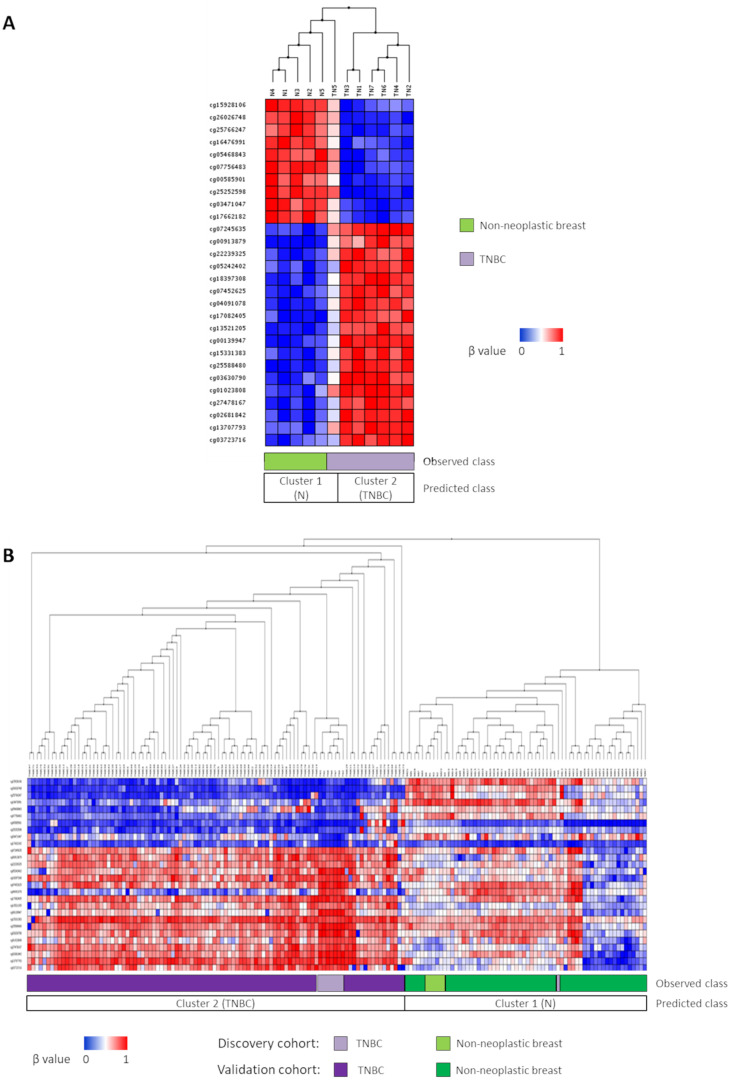
TNBC predictor signature. (**A**) A class prediction method provided a signature of 35 differentially methylated probes between non-neoplastic breast (N, *n* = 5) and TNBC samples (TN, *n* = 7) with a prediction error rate of 0.083. (**B**) Signature extended to larger independent cohorts of additional 58 non-neoplastic (GSE88883 and GSE74214) and 93 TNBC samples (GSE78751 and GSE78754) yielded an error rate of 0.006. Light colours represent discovery cohort. In both panels, the class is predicted based on the cluster segregation depicted in the dendrogram.

To identify potential diagnostic biomarkers for TNBC, a signature of DMPs with class prediction ability between non-neoplastic breast (*n* = 6) and TNBC (*n* = 8) tissues was generated by comparing their DNA-methylation patterns. Based on our previous results [28], two samples, one non-neoplastic and one tumoural, had DNA methylation profiles quite different from those of the remaining samples in their groups, and were excluded from this analysis. Thus, the class-predictive signature with the minimum prediction error rate (0.083) that we found between 7 TNBC and 5 non-neoplastic samples was composed of 35 DMPs (Supplementary Table S2). Indeed, this signature accurately classified six out of seven TNBC and five out of five non-neoplastic breast samples in the TNBC and non-neoplastic groups, respectively (sensitivity = 83%; specificity = 100%) (Figure 1A).

In order to test the robustness of the class prediction model, our predictor signature was extended to larger cohorts of 58 non-neoplastic breast (GSE88883 and GSE74214) and 93 TNBC (GSE78751 and GSE78754) samples, whose methylation data were publicly available. Since the β value of one of the probes (cg15555527) could not be retrieved from those samples, the signature was then composed of 34 probes. Importantly, we found that this 34-predictor signature consistently made all TNBC samples cluster together, regardless of the data set to which they belonged (Figure 1B). Thus, the discriminative power of the diagnostic signature raised, categorizing 62 out of 63 non-neoplastic breast samples and 100 out of 100 TNBCs as the non-neoplastic and TNBC groups, respectively, and therefore, yielding a sensitivity of 98.4% and a specificity of 100% (error rate = 0.006) (Figure 1B).

**Figure 2:**
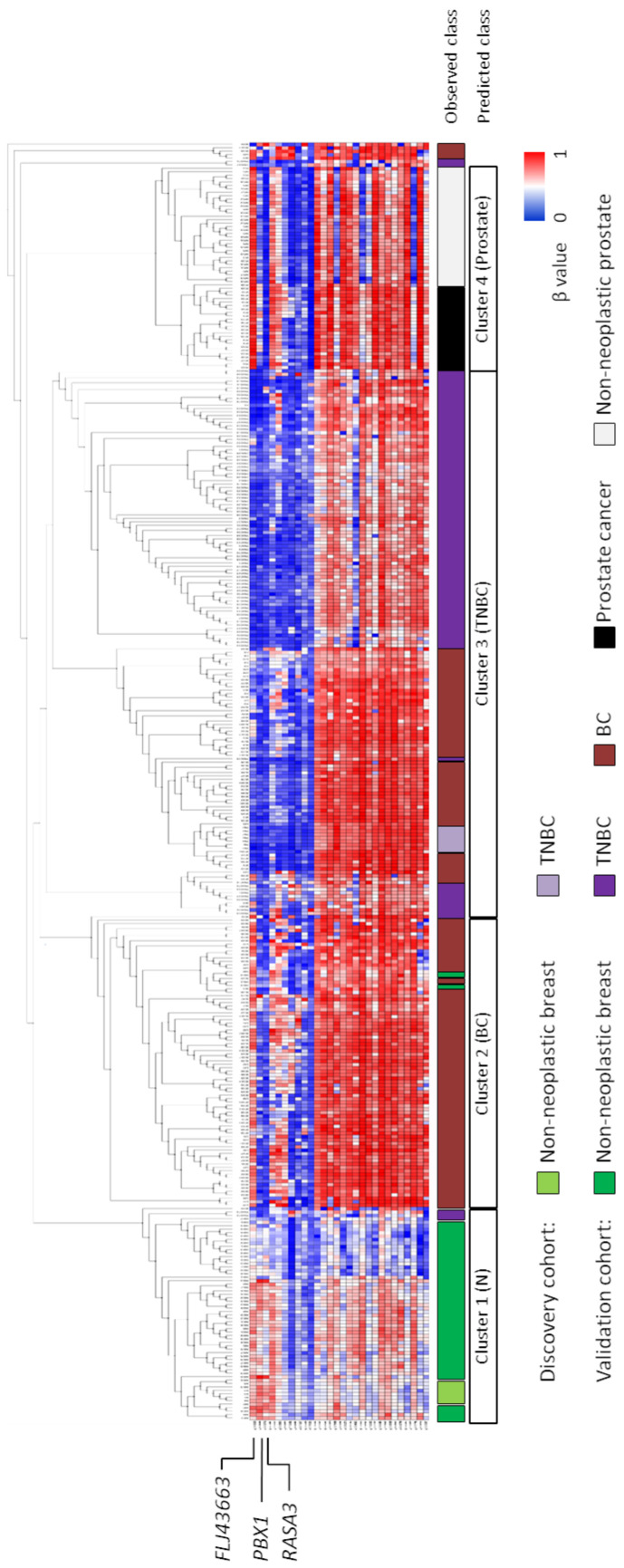
Robustness of our DNA methylation signature as predictor for TNBC diagnosis. Methylation levels of the 34 predictor probes were explored in independent large cohorts from public repositories of 63 non-neoplastic breast (GSE141338, GSE88883, and GSE74214), 100 TNBC (GSE141338, GSE78751, and GSE78754), 150 BC (GSE141338, and GSE72245), and 60 prostate-derived tissues (GSE76938). Light colours represent discovery cohort. The class is predicted based on the cluster segregation depicted in the dendrogram. Genes corresponding to probes with the highest methylation fold change (|Δβ|) between TNBC and non-neoplastic mammary samples, and selected for further validation are pointed out.

The methylation levels of these 34 probes were also explored in a total of 150 samples diagnosed with BC of confirmed non-TNBC subtype (GSE141338) and unknown subtype (GSE72245). Above that, 30 non-neoplastic and 30 tumour prostate samples (GSE76938) were included as unrelated tissues to ensure that the observed differences in breast tissues were not due to slight differences in distinct subtypes from the same mammary origin. With the exception of a first small set of five non-TNBC cases and a second one with only two TNBC cases that segregated soon from the remaining patients, all samples derived from prostate tissues were, as expected, the least closely related to the breast ones (Figure 2). Regarding BC tissues, more than half of them clustered together and separated from non-neoplastic samples. Interestingly, a subset of BC cases of unknown subtype was located within the TNBC cluster. This finding could suggest their belonging to the TNBC subtype, but it cannot be confirmed, because information regarding molecular subtypes of BC from these public data sets was not available. Taken together, these results strengthen the robustness of our methylation signature to predict the TNBC diagnosis.

### 3.2. Methylation Status of FLJ43663, PBX1, and RASA3 in Breast Tissue and Plasma

**Figure 3:**
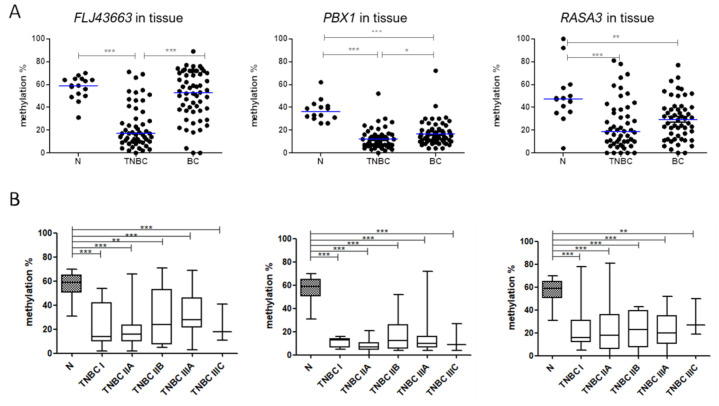
Methylation status of the *FLJ43663*, *PBX1,* and *RASA3* genes in breast tissues. (**A**) Methylation levels of these genes were measured by pyrosequencing in FFPE samples from 16 non-neoplastic breast tissues (N), 51 TNBC, and 57 BC tissues other than TNBC subtype. The horizontal blue lines represent the median of the series. (**B**) Methylation percentages of the three selected genes in 16 non-neoplastic breast tissues (N), and tumours from patients with TNBC at stage I (*n* = 8), stage IIA (*n* = 21), stage IIB (*n* = 6), stage IIIA (*n* = 8), and stage IIIC (*n* = 3) (*, *p* < 0.05; **, *p* < 0.01; ***, *p* < 0.001).

Among the 34 predictor probes with the highest |Δβ| values in TNBC comparing to non-neoplastic tissues (Supplementary Table S2), three were selected to validate their usefulness as biomarkers for TNBC diagnosis: cg15928106, cg2626748, and cg16476991, which recognised *FLJ43663*, PBX Homeobox 1 (*PBX1*), and RAS P21 protein activator 3 (*RASA3*) genes, respectively (Figure 2). To do this, we measured by pyrosequencing the methylation levels of the genomic region recognized by each probe in a series of FFPE samples from 51 TNBC patients and 16 non-neoplastic breast tissues. We confirmed that TNBC tumours had significantly lower methylation levels of these selected genes than non-neoplastic samples (*p* ≤ 0.001) (Figure 3A). Moreover, those tumours even showed significantly lower methylation compared to other BC subtypes (*n* = 57, *p* ≤ 0.02) (Figure 3A). The optimal cut-off value distinguishing statistically the hypomethylated and hypermethylated status of each selected gene was estimated by ROC curve analysis (56% methylation for *FLJ43663*, 31% methylation for *PBX1*, and 36% methylation for *RASA3*). Based on these cut-offs, the simultaneous hypomethylation of the three selected probes can distinguish TNBC from non-neoplastic tissue with an error rate of 0.25. Therefore, the hypomethylation of *FLJ43663*, *PBX1,* and *RASA3* could be a specific epigenetic biomarker predictive of TNBC diagnosis. Interestingly, the hypomethylation of these genes was maintained across all stages of TNBC, with no differences between them, but significantly lower than non-neoplastic breast tissues (*p* < 0.05), even at early stages (Figure 3B).

**Figure 4:**
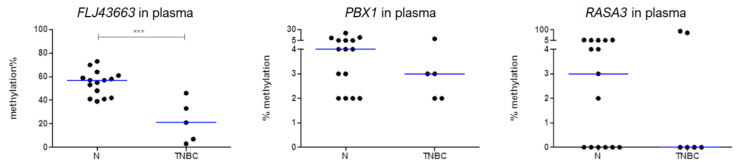
Methylation percentage of the *FLJ43663*, *PBX1,* and *RASA3* genes in total circulating cell-free DNA. Methylation levels of these genes were measured by pyrosequencing in plasma samples from 15 healthy women (N) and 6 TNBC patients. The horizontal blue lines represent the median of the series (***, *p* < 0.0001).

In order to determine whether these potential diagnosis biomarkers could also be detected by non-invasive methods, the methylation levels of *FLJ43663*, *PBX1,* and *RASA3* in total cfDNA in plasma were preliminarily studied in a small series of six TNBC patients and 15 age-matched healthy women. As in tumour tissues, TNBC patients also displayed in cfDNA lower methylation levels of the three selected genes than did healthy women, reaching *FLJ43663* to statistical significance (*p* < 0.0001) (Figure 4).

## 4. Discussion

Even if TNBC presents the most aggressive clinical behaviour among BC subtypes [6,38], its clinical management is characterized by the lack of biomarkers. Currently, in routine practice, a patient with clinical manifestations is diagnosed with TNBC when no expression of ER, PR, and HER2 is detected in her tumoural biopsy by immunohistochemistry. However, preanalytic variables, different thresholds for negativity and diverse interpretation criteria, along with interlaboratory discordances, persist and may generate inaccurate results [10,39]. For instance, the 2010 American Society of Clinical Oncology and the College of American pathologist guidelines suggest that up to 20% of ER and PR test results are false negative or false positive, and these misinterpretations can lead to the administration of ineffective therapies to inaccurately diagnosed patients [14,40]. Therefore, there is an unmet need for a robust method, complementary to immunohistochemisty, for TNBC diagnosis. 

Since some genes seem to acquire a tissue-specific DNA methylation pattern [41], and aberrant DNA methylation is a common and early event in tumourigenesis [42,43], this epigenetic alteration is deemed as a promising biomarker for cancer diagnosis [44]. As DNA methylation is a stable modification, it can be profiled using small amounts of routinely collected DNA samples from biopsies, or even DNA released by tumour cells into biological fluids, such as peripheral blood, which can be detected through non-invasive methods [45,46]. All these features make DNA methylation an easily and readily applicable tool useful for the clinical practice.

In the last decades, whole genome strategies have dramatically expanded the identification of aberrantly methylated genes [15,47], allowing systematic approaches that seek for maximum accuracy by the generation of diagnostic signatures. Thus, in addition to classical biomarkers of diagnosis based on single methylated genes [43,48], some specific DNA-methylation-based signatures have been described for the detection of several tumours, such as prostate cancer [49,50], hepatocellular carcinoma [51], and glioma [52]. However, virtually no diagnostic biomarker or signature based on DNA methylation has been proposed for TNBC. Indeed, few methylation studies have been carried out in this specific BC subtype, and hardly any reports have profiled DNA methylome in TNBC. Those investigations have focused on TNBC subclassification, comparing DNA methylome among TNBC samples [24,25], or the identification of DNA methylation aberrations as potential prognosis biomarkers [26,27]. Only Stirzaker et al. [27] have used 282 TNBC-specific probes to classify TNBC and non-TNBC; however, this signature displayed a lower sensitivity compared with ours (72% vs 83%). Moreover, the authors compared TNBC samples with “normal” adjacent-to-tumour tissues, but, as we [28,53] and others have demonstrated [54,55,56,57,58,59,60], the latter should not be considered as such a good control group due to field cancerization phenomenon, by which adjacent-to-tumour breast tissue appears histologically normal, but contains changes in DNA methylation that may contribute to tumour initiation [54].

In the present study, we identified a novel DNA methylation signature with a high predictive power for distinguishing TNBC from purely non-neoplastic breast tissue and even from other BC subtypes. To the best of our knowledge, this is the first study focusing on DNA methylome aiming to generate a diagnostic signature in TNBC. Three probes with the highest Δβ values between TNBC and non-neoplastic samples, located in the *FLJ43663*, *PBX1,* and *RASA3* genes, were selected from the predictive signature and further validated. First, *FLJ43663* is a long non-coding RNA also known as long intergenic non-protein-coding RNA p53-induced transcript (LINC-PINT). Several studies have demonstrated the involvement of its down-regulation in cancer progression and tumour malignancy, and therefore, it has been suggested that it could act as a tumour suppressor in different types of cancer, such as lung, ovarian, pancreatic, and breast carcinomas, among others [61,62,63,64,65]. *FLJ43663* has been also proposed as a diagnostic biomarker for non-small cell lung and pancreatic cancer [63,65]. Above that, a recent investigation illustrated a tumor suppressor role of *FLJ43663* in sensitizing TNBC to chemotherapies [66]. In our research, the hypomethylation of *FLJ43663* in TNBC compared with healthy women was confirmed for the first time. Further research is needed to depict the influence of this epigenetic aberration in TNBC tumourigenesis. Second, the protein encoded by the *RASA3* gene functions as a negative regulator of the Ras signalling pathway [67]. Recently, *RASA3* hypomethylation has been identified as a potential mechanism for hepatocellular carcinoma development, and therefore, as a useful biomarker for early detection [68]. Accordingly, here, we report the potential usefulness of *RASA3* hypomethylation as a part of a signature for TNBC diagnosis. Lastly, the *PBX1* gene encodes a nuclear protein that belongs to the PBX (Pre-B cell leukaemia transcription factor) homeobox family of transcriptional factors and is involved in a chromosomal translocation in human pre-B cell acute lymphoblastic leukaemia [69]. Its high expression has been related to BC aggressiveness [70], and proposed as a biomarker of poor prognosis in ER-positive BC [71], and also in ER-negative BC when coexpressed with *EMP2* [72]. However, the epigenetic status of *PBX1* in any BC has not been elucidated yet. Here, we provide the first description of *PBX1* hypomethylation in TNBC compared to non-neoplastic breast tissue and other BC subtypes, which could explain its already described overexpression.

Of note, besides the hypomethylation of *FLJ43663, PBX1,* and *RASA3* in TNBC compared to non-neoplastic breast samples, we also found that these epigenetic biomarkers were present at any stage of the disease, even at the earliest ones, which could make them potential candidates for TNBC screening, if these findings are confirmed in larger series of patients. In this regards, non-invasive approaches for the detection of cancer biomarkers, such as liquid biopsy, have acquired enormous momentum in the last few years [73]. Specifically, cfDNA in plasma from cancer patients has been explored as a diagnostic material, since it can be detected at early stages of the disease [74], and consistently mimics DNA methylation signatures present in tumoural DNA [74,75,76,77]. Consequently, some tumour-specific methylated genes in cfDNA have been described to have potential value for early detection of cancer, particularly in BC [21,22,23]. However, these promising evidences regarding altered DNA methylation in cfDNA have not been studied in TNBC yet. Newly, our group has reported the hypomethylation of the *ADAM12* gene as the first change in methylation detected specifically in plasma of patients with TNBC comparing to age-matched healthy women [28]. In view of the usefulness of cfDNA as an informative material for the identification of epigenetic biomarkers with clinical value, here, we report the methylation status of the three selected probes in cfDNA. Even if the trend to lower methylation levels of *PBX1* and *RASA3* did not reach statistical significance, probably due to the limited number of samples, we demonstrate the significant hypomethylation of *FLJ43663* also in cfDNA from TNBC patients compared to plasma from healthy women, mirroring the epigenetic alteration found in the tumoural tissue. This preliminary evidence opens the door to further investigations in larger and independent series of patients to confirm if our DNA methylation-based diagnostic signature could be also assessed not only in the tumour sample obtained by routine biopsy, but also in a non-invasive manner in plasma of patients with TNBC, even at earliest stages of the disease.

In summary, we identified a novel DNA methylation signature with predictive value for TNBC diagnosis, regardless of the stage, and proposed one surrogate, *FLJ43663*, whose hypomethylation may be detected in both tumour tissue and plasma from TNBC patients with potential predictive purposes for diagnosis.

## Figures and Tables

**Table 1:** Primer sequences used in PCR and pyrosequencing, resulting amplicon size, and specific melting temperatures (Tm). Primers were designed using PyroMark Assay Design 2.0 software (Qiagen, Hilden, Germany). Btn, biotin.

Gene	Forward Primer (5′-3′)	Reverse Primer (5′-3′)	Sequencing Primer (5′-3′)	Size (bp)	Tm (°C)
***FLJ43663***	TTGTTTTGAAGGTGGTAAATTAGATT	Btn-ATCCCCTTAATAAATAAAACTACACATC	AAGGTGGTAAATTAGATTTT	108	58
***PBX1***	AGAAGGAAGTGGTTTTGTTTAGA	Btn-CTATCAACCAAAAAAAACAAACAATAACA	TTGTTTAGAGGTTATATTTAGTG	83	60
***RASA3***	ATAGATGGGGAGATTGAGGTT	Btn-ATCTTCAAACCAAACCCAAAAACTCAATAA	AGTTGTGAGTTTTAGTTTAG	118	60

## Data Availability

The data sets analysed in this study are publicly available in the Gene Expression Omnibus repository under the following accession numbers: GSE141338 (discovery series of 5 non-neoplastic and 7 TNBC samples), GSE88883 and GSE74214 (58 non-neoplastic breast samples), GSE78751 and GSE78754 (93 TNBC), GSE141338 and GSE72245 (150 BC), and GSE76938 (60 prostate-derived tissues).

## Supplementary Materials

The following are available online at https://www.mdpi.com/article/10.3390/biomedicines9101394/s1, Table S1: Pathological and clinical characteristics of TNBC patients from the FFPE cohort used for validation (*n* = 51), Table S2: Description of the 35 differentially methylated probes with predictive value of TNBC diagnosis.
