# Supplementary material for: A DNA Methylation-Based Gene Signature Can Predict Triple-Negative Breast Cancer Diagnosis
Source: Biomedicines. 2021 Oct 4;9(10):1394. doi: 10.3390/biomedicines9101394 (PMC8533184; doi:10.3390/biomedicines9101394)
Supplement: Supplementary file 1 [file biomedicines-09-01394-s001.zip › biomedicines-1325831-supplementary.pdf]

Table S1. Pathological and clinical characteristics of TNBC patients from the FFPE cohort used for validation ($n = 51$).

Feature	Frequency (%)
**Age** (years)	
Mean (range)	59.26 (31–81)
**Tumour size** (cm)	
Mean (range)	2.2 (0.3–10.0)
**Lymph node involvement**	
No	29/51 (57.0)
Yes	21/51 (41.0)
n.a.	1/51 (2.0)
**Stage**	
I	11/51 (21.0)
IIA	22/51 (43.0)
IIB	6/51 (12.0)
IIIA	8/51 (16.0)
IIIC	3/51 (6.0)
n.a.	1/51 (2.0)
**Progression-free survival** (months)	
Mean (range)	81.7 (10.3–216.9)
**Progression**	
No	34/51 (66.6)
Yes	16/51 (31.4)
n.a.	1/51 (2.0)
**Overall survival** (months)	
Mean (range)	89 (10.3–216.9)
**Outcome**	
Exitus	15/51 (29.0)
n.a.	1/51 (2.0)
**Chemotherapy**	
No	8/51 (16.0)
Yes	41/51 (80.0)
n.a.	2/51 (4.0)
**Radiotherapy**	
No	11/51 (21.0)
Yes	38/51 (74.0)
n.a.	2/51 (4.0)

n.a.: non-available data.

Table S2. Description of the 35 differentially methylated probes with predictive value of TNBC diagnosis. Each Δβ value was calculated by subtracting the mean β value of TNBC (TN) samples minus the mean β value of samples from non-neoplastic breast samples (N), patients with other BC subtypes (BC), or each of them: Luminal A (LA), Luminal B/HER2 negative (LB), Luminal B/HER2 positive (TN) and HER2 (H). Probes are sorted by the Δβ in TNBC minus Δβ in N.

Probe Name	Gene Name	Δβ (TN-N)	Δβ (TN-BC)	Δβ (TN-LA)	Δβ (TN-LB)	Δβ (TN-LH)	Δβ (TN-H)
cg15928106	*FLJ43663*	-0.39	-0.38	-0.42	-0.46	-0.39	-0.24
cg05908105	*NET1*	-0.37	-0.07	-0.08	-0.02	0.00	-0.17
cg26026748	*PBX1*	-0.35	-0.05	-0.03	-0.10	-0.02	-0.04
cg16476991	*RASA3*	-0.34	-0.19	-0.20	-0.15	-0.22	-0.19
cg25766247		-0.32	0.00	-0.02	0.03	0.01	-0.03
cg05468843	*IL10RA*	-0.30	-0.41	-0.38	-0.44	-0.44	-0.38

cg09323975		-0.25	-0.14	-0.13	-0.15	-0.11	-0.15
cg07756483	*KCNN4*	-0.22	-0.32	-0.34	-0.36	-0.40	-0.17
cg00585901	*EFNA4*	-0.17	-0.20	-0.21	-0.19	-0.25	-0.14
cg03471047		-0.14	-0.04	-0.01	0.01	-0.05	-0.09
cg25252598		-0.14	-0.09	-0.13	0.00	-0.16	-0.07
cg01794555	*LCP1*	-0.13	-0.07	-0.06	-0.13	-0.08	-0.03
cg17662182		-0.09	-0.02	-0.01	-0.01	-0.01	-0.06
cg07245635	*S100A4*	0.12	-0.03	-0.04	-0.03	-0.02	-0.02
cg00534684	*PTAFR*	0.13	0.01	0.01	0.00	0.01	0.01
cg00913879	*DNAH17*	0.13	-0.04	-0.06	-0.05	-0.05	-0.01
cg14379490	*FAM120A*	0.14	0.02	0.01	0.01	0.03	0.05
cg05242402	*MICALL2*	0.16	0.03	0.03	0.02	0.01	0.06
cg04091078	*SLCO1C1*	0.17	0.04	0.03	0.09	0.03	0.02
cg18397308	*C19orf50*	0.17	0.00	0.00	-0.01	0.00	0.02
cg22239325	*MBP*	0.17	0.07	0.02	0.09	0.15	0.05
cg07452625	*SPG7*	0.17	0.00	0.00	-0.04	-0.01	0.04
cg25590335	*PKN1*	0.18	0.00	0.00	-0.02	0.01	0.01
cg17082405	*PLEC1*	0.18	0.00	-0.01	-0.01	-0.01	0.04
cg13521205	*FAM129B*	0.19	-0.02	-0.03	-0.05	-0.04	0.03
cg15331383	*RPTOR*	0.20	-0.03	-0.01	-0.07	-0.05	0.01
cg27478167	*HEATR2*	0.20	-0.01	-0.01	-0.05	-0.02	0.02
cg25588480	*MINK1*	0.20	0.01	0.01	0.00	0.00	0.04
cg00139947	*TOP1MT*	0.20	0.02	0.02	-0.01	0.00	0.05
cg03630790	*TGFBR2*	0.20	0.02	0.04	0.00	-0.01	0.04
cg02681842	*PLEC1*	0.23	0.01	0.02	-0.03	-0.02	0.06
cg01023808	*TECPR1*	0.23	0.01	0.02	0.00	0.00	0.01
cg13707793	*CXXC5*	0.27	0.04	0.02	0.10	0.00	0.04
cg03723716	*PPP2R1B*	0.29	0.08	0.05	0.13	0.02	0.14
cg15555527	*ANTXR2*	0.36	NA	NA	NA	NA	NA
